# The effectiveness of *Annona muricata* for improving induced oral candidiasis in gamma-irradiated rats

**DOI:** 10.1186/s12906-026-05399-y

**Published:** 2026-05-14

**Authors:** Mostafa A. Bakr, Nadia A. Elkady, Amr H. Rasmy

**Affiliations:** 1 Health Radiation Research Dept., National Center for Radiation Research and Technology, Egyptian Atomic Energy Authority, Cairo, Egypt; 2 Microbiology Dept., Faculty of Science, Ain Shams University, Cairo, Egypt

**Keywords:** *Annona muricata*, Nystatin, Oral candidiasis, Gamma-irradiation, Antifungal, Tongue, *Candida tropicalis*

## Abstract

**Background:**

Radiotherapy is an appropriate treatment for malignancies but can impair antifungal defense, promoting oral candidiasis. *Candida tropicalis* is of particular concern in immunocompromised patients and its management remains challenging. This study evaluated the antifungal efficacy of *Annona muricata* and its potential synergistic effect with nystatin against induced oral candidiasis in gamma-irradiated rats.

**Methods:**

Forty male albino rats were assigned to five groups: R (irradiated), RC (irradiated infected), RCN (treated with nystatin), RCA (treated with *A. muricata*), and RCNA (treated with both therapies). Antifungal activity against *C. tropicalis* isolated from human oral lesions was assessed. Infection was introduced on the dorsal tongue surface, and treatment was initiated 48 h after inoculation. Rats were sacrificed on days 7 and 10. Body weight, tissue fungal burden, histopathological changes, and scanning electron microscopic findings were evaluated.

**Results:**

*A. muricata* and nystatin reduced fungal burden and improved mucosal healing, with nystatin performing slightly better as a single treatment. However, their combined use produced the greatest overall improvement in morphology, fungal counts, tissue integrity, and microscopic findings. Untreated infected group showed persistent severe lesions and high fungal load. These results demonstrate a more pronounced effect when both therapies are applied together.

**Conclusions:**

*A. muricata* may exert antifungal and tissue-protective effects in a gamma-irradiated rat model of oral *C. tropicalis* infection. Its combination with nystatin may further enhance fungal clearance, improve mucosal integrity, and better maintain body weight compared with either treatment alone.

## Background

Radiotherapy (RT) is a standard and effective therapy for the management of malignant tumours that may be used either alone or combined with surgery and chemotherapy [1]. Nevertheless, it leads to various side effects to surrounding healthy tissues including xerostomia, tooth decay, as well as compromised oral hygiene [2].

Human bodies are hosts for millions of microorganisms. *Candida* species are type of yeasts usually found in the normal human flora. They inhabit several parts of human body especially mucosal surfaces as vagina, gastrointestinal tract and oral cavity [3, 4]. *Candida* species normally exist in almost 50% of the oral cavities of healthy persons [5]. Local or systemic impairment of the antifungal defense mechanism of human body, enhances the overgrowth of *Candida* species and the development of candidiasis especially in immunocompromised patients as head and neck cancer patients [3].

Oral candidiasis can display different manifestations when *Candida* combines with immunosuppression conditions as RT [6]. RT can aggravate oral candidiasis thus more virulent *Candida* strains can develop in radiotherapeutic patients [7]. Consequently, the emergence of antifungal resistance can occur in those immunocompromised patients [3].

An epidemiological trend indicates a growing prevalence of non-albicans *Candida* (NAC) species, particularly *C. tropicalis*, *C. parapsilosis*, and *C. glabrata*. Among these, *C. tropicalis* is of special concern in tropical regions and among immunocompromised individuals, such as patients with neoplastic diseases, owing to its strong adhesion properties and robust biofilm forming ability [8, 9]. Jain et al. [10] reported a marked increase in *Candida* colonization among oral cancer patients undergoing radiotherapy and/or chemotherapy, with *C. tropicalis* being more prevalent (42.85%) than *C. albicans* (14.28%). Similarly, Kermani et al. [11] also identified *C. tropicalis* among oral isolates from radiotherapeutic patients, confirming its persistence alongside *C. albicans* in this clinical context.

The treatment of mucosal infections caused by *Candida* and the elucidation of the disease process have proven challenging. Nystatin is an effective broad-spectrum antifungal drug that is frequently used to treat superficial candidiasis [12]. Alternative medicines and natural remedies have become more interesting for researchers owing to their low costs and reduced side effects [13]. *Annona muricata* (*A. muricata*, soursop or graviola) is a tropical fruit species that has shown potential in the treatment of some diseases as diabetes, parasitic infections and cancer [14]. Additionally, its extract is considered a promising antifungal agent for infections caused by *Candida *[15].

*A. muricata* exhibits well-documented antifungal, antioxidant, and gamma-irradiation-protective properties. Previous studies have demonstrated potent antifungal activity of *A. muricata*, as evidenced by a significant inhibition of fungal growth, reduced cell density, and disruption of plasma membrane and cell wall integrity, leading to decreased fungal viability [15]. It also effectively suppressed the growth and adhesion of multidrug-resistant *Candida albicans* (*C. albicans*), an effect associated with reduced carbohydrate, protein, and extracellular DNA components within the biofilm matrix [16]. Notably, the combination of *A. muricata* with fluconazole enhanced the inhibition of *C. albicans* biofilm formation [17]. In vitro studies further confirmed its broad antimicrobial and antifungal activity against several oral pathogens, including *C. albicans *[18].

In vivo evidence supports the therapeutic potential of *A. muricata* in vulvovaginal candidiasis, where treatment resulted in effective fungal clearance, reduced tissue fungal burden, and attenuation of mucosal inflammation [16, 19].

Beyond its antifungal effects, *A. muricata* has demonstrated radioprotective activity in multiple in vivo models including lung, kidney, skin, ileum, liver, and brain tissues. These effects are largely attributed to its antioxidant capacity, which mitigates oxidative stress and reduces radiation-induced tissue damage during radiotherapy [20–25].

Additionally, *A. muricata* exhibited antitumor activity by reducing tumor volume, prolonging survival in Ehrlich ascites carcinoma-bearing mice, and exerting selective cytotoxicity against several cancer cell lines. It also attenuated radiation- and chemotherapy-related toxicity by preserving antioxidant defenses and limiting oxidative damage [20, 26].

The study of experimentally induced oral candidiasis is useful to clarify the etiopathogenesis of this condition and search for new therapeutic options. Therefore, the present study was designed to investigate the antifungal efficacy of *A. muricata* and its potential synergistic effect with nystatin against induced oral candidiasis in gamma-irradiated rats. The null hypotheses proposed that *A. muricata* exerts no significant antifungal effect against induced oral candidiasis in gamma-irradiated rats, and that the combined administration of *A. muricata* and nystatin yields no significant enhanced antifungal effect.

## Methods

**Fig. 1:**
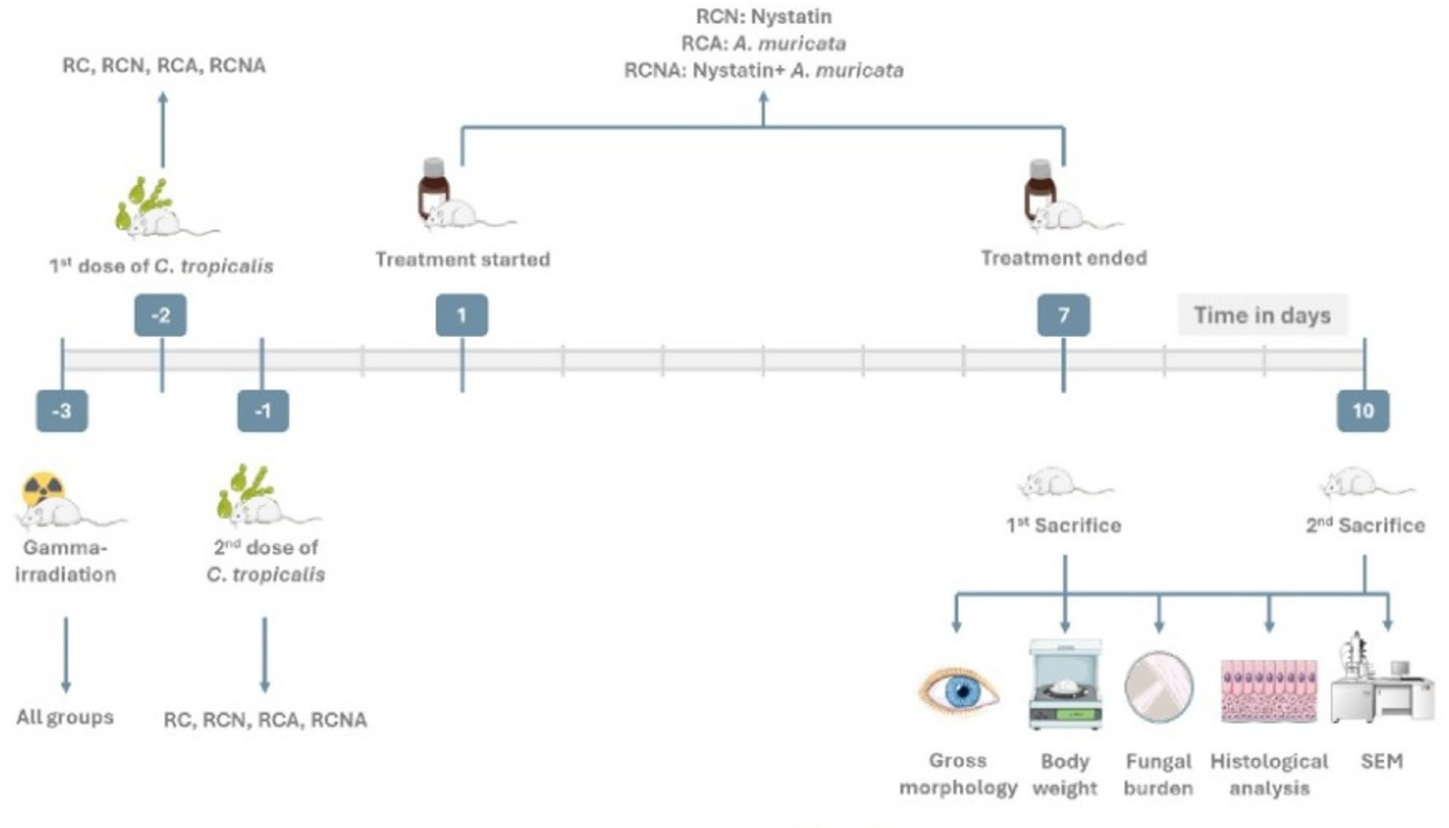
Experimental design. https://smart.servier.com/ & https://bioicons.com/

This experimental study was conducted to evaluate the antifungal efficacy of *Annona muricata* (*A. muricata*) and its potential synergistic effect with nystatin against induced oral candidiasis in gamma-irradiated rats. Multiple assessment methods, including morphological, body weight, microbiological, histopathological, and scanning electron microscopic analyses were employed to evaluate treatment outcomes over specified time points. Workflow of the experiment is illustrated in Fig. 1.

### Ethics statement and animal care

The experiment was conducted according to the protocol approved by the Research Ethics Committee of the National Center for Radiation Research and Technology (REC-NCRRT), Egyptian Atomic Energy Authority, no. 49 A/23.

### Sample size calculation

According to the previous study conducted by Abd El-Monem & Elwakeel [27], minimum total sample size of 40 samples was sufficient to detect the effect size of 0.27, with a power (1-β = 0.90) at a significance probability level of *p* ≤ 0.05, to verify the aim of our study. According to sample size calculations, there is a 90% chance of correctly rejecting the null hypothesis of no significant effect if each group represented by eight samples. The sample size was calculated according to G*Power software version 3.1.9.7.

### Animals and grouping

Forty adult male albino rats (190 ± 10 g) approximately 8 weeks old were obtained from the breeding unit of the National Center for Radiation Research and Technology (NCRRT), Egyptian Atomic Energy Authority, Cairo, Egypt. The animals were acclimatized to laboratory environment for two weeks before the beginning of the experiment. They were kept in polypropylene/stainless steel cages having dimensions of 54, 37 and 27 cm, and submitted to suitable ventilation, humidity (max 55%), controlled temperature (25 max 28 °C) and light/dark regime (12 h of light/dark cycle) with access to pellet diet and drinkable water *ad libitum*. Cage bedding was replaced and cages were cleaned daily, with disinfection performed at three-day intervals. The investigators and the workers of the animal house of the National Center for Radiation Research and Technology (NCRRT), Egyptian Atomic Energy Authority, Cairo, Egypt were responsible for the care of the animals; however, euthanasia was the investigators responsibility. No humane ends were expected during the study.

Animals were randomly divided into five groups (*n* = 8): R: rats were subjected to gamma-irradiation only, RC; rats were subjected to gamma-irradiation followed by the induction of experimental candidiasis, RCN; oral candidiasis was induced after gamma-irradiation followed by the treatment with nystatin, RCA; oral candidiasis was induced after gamma-irradiation followed by the treatment with *A. muricata*, RCNA; infected gamma-irradiated rats were treated with nystatin along with *A. muricata*.

### Gamma-irradiation

Rats of all groups were completely immobilized inside a special shield then subjected to a single dose of localized (cranium) gamma-irradiation at dose of 20 Gy [28] and dose rate of 0.6144 KGy/hr at the time of the study, using the ^60^Co Gamma Cell (220, India) at the National Center for Radiation Research and Technology (NCRRT), Egyptian Atomic Energy Authority, Cairo, Egypt. Before the localized irradiation, each rat was intra-peritoneally anesthetized by an anaesthetic combination of 90 mg/kg ketamine and 10 mg/kg xylazine 2% (2:1) (0.12 ml/100 g body weight) [29].

### Preparation of Candida tropicalis inoculum

The antifungal efficacy of *A. muricata* and nystatin was tested against *Candia tropicalis* (*C. tropicalis*) isolated from erythematous oral lesions in a patient after obtaining an informed donor consent. The strain was cultured and maintained by the Microbiology Dept., Faculty of Science, Ain Shams University, Cairo, Egypt. *C. tropicalis* strain was initially identified on chromogenic agar media (Brilliance™ Candida Agar) and the identification was further confirmed using Matrix-Assisted Laser Desorption Ionization-Time of Flight Mass Spectrometry Analysis (MALDI-TOF MS) (Children’s Cancer Hospital Egypt 57357, Cairo, Egypt). To induce candidiasis in animals, *C. tropicalis* was grown on Sabouraud’s dextrose agar plates (Himedia, Mumbai, Maharashtra, India) at 37 °C for 24–48 h. After incubation, a colony of the strain was transferred into 10 ml of yeast extract peptone-dextrose (YPD) broth (Difco Laboratories, Detroit, Mich.) and incubated in a 30 °C shaker at 180 rpm for 15–18 h. Yeast cells were harvested and washed with sterile phosphate buffered saline (PBS) twice. Cells were then re-suspended in 10 ml of PBS and serially diluted and adjusted to a concentration of 3 × 10^8^ CFU/mL at optical density of 300 nm (OD300) measured using a spectrophotometer [30].

### Induction of experimental oral candidiasis

Rats of all groups were given 0.1% solution of tetracycline hydrochloride in drinking water. This management was initiated 7 days before the inoculation of *Candida* suspension and was maintained up to the end of the experiment to facilitate the persistent oral carriage of *C. tropicalis *[31] except the day of gamma-irradiation. At the next day of gamma-irradiation [32], the dorsum of tongues of infected groups were lightly and superficially scratched so that neither trauma nor bleeding occurred [33].

For the inoculation of this suspension, rats were intra-peritoneally sedated by an anaesthetic combination of 90 mg/kg ketamine and 10 mg/kg xylazine 2% (2:1) (0.12 ml/100 g body weight) [29]. Rats were monitored for signs of awaking including blinking of the eyes and movement of the extremities. The introduction of a suspension of *C. tropicalis* containing 3 × 10^8^ CFU/ml was performed for two consecutive days. Infection was impregnated on the dorsal surface of the rats’ tongues by gently swabbing the epithelial tissue with sterile laboratory swabs saturated with 0.1 ml of a freshly prepared suspension [30]. After the induction of candidiasis, swabs from the tongue surfaces were collected and cultured on chromogenic agar media (Brilliance™ Candida Agar) to confirm the onset of the infection. Treatment by nystatin and/or *A. muricata* started for all rats of RCN, RCA and RCNA groups 48 h after the last induction dose [34].

### Nystatin application

A dose of 0.5 ml/rat/day of Mycostatin oral suspension (GlaxoSmithKline, Elsalam City, Cairo, Egypt) containing 100,000 IU/ml nystatin, was topically applied to the dorsal surface of the rats’ tongues of groups RCN and RCNA for seven consecutive days using a one ml syringe [30, 33].

### *Annona muricata* application

*A. muricata* suspension at a dose of 400 mg/kg bodyweight was orally introduced to rats of RCA and RCNA groups [35]. Graviola capsules (Bio Nutrition, Inc., NY, USA) containing 1500 mg *A. muricata* were used. The powder in each capsule was dissolved in 20 ml distilled water just before use to obtain a solution of a concentration of 75 mg/ml [27]. Each rat received one ml of the forementioned solution by oral gavage for seven consecutive days [16].

After the last treatment dose, half of the animals from each group were euthanized at the first time point (day 7) by an intra-peritoneally injected over-dose anaesthesia (Ketamine) [30], while the remaining animals were maintained for a further 3-day observation period to assess potential infection relapse then euthanized at the second time point (day 10). Tongue specimens were excised from euthanized animals in each group for subsequent analyses.

### Morphological examination and body weight measurements

Gross morphological assessment of the rats’ tongues was conducted on days 7 and 10 to evaluate the presence and extent of fungal infection. Furthermore, body weight was monitored and recorded daily starting from day one throughout the study period.

### Quantification of fungal burden and histopathological analysis

For determination of the fungal burden, part of each excised tongue was placed on a pre-labelled petri dish on ice until the weight is recorded. Each tissue sample was transferred to 5 ml of 0.85% saline and homogenized for approximately 10–20 s. Ten-fold serial dilutions of the homogenized tissues were plated on Sabouraud’s dextrose agar (SDA) containing 100 µg/ml chloramphenicol and incubated at 37 °C for 24 h. Grown yeast colonies were counted, and tissue burden was expressed as CFU/gm. Counts were log10-transformed for analysis, and to avoid undefined values for zero counts, a value of 1 was assigned prior to transformation [3].

Moreover, another part of the excised tongue was immediately fixed in 10% neutral buffered formalin and embedded in paraffin. Sections of 5-µm thickness were cut and submitted to staining with hematoxylin & eosin (H&E) for histopathological observations as well as periodic acid-Schiff reagent (PAS) to detect fungal elements in tissue. The morphological changes were evaluated according to the presence of yeast cells, the organization of the epithelial layer, and the presence of inflammatory cells in the connective tissue. Representative images of examined tissue sections were captured using Leica DM3000 microscope equipped with a Leica K3C digital camera and Leica Application Suite X (LAS X) software (Leica Microsystems CMS GmbH, Wetzlar, Germany).

### Scanning electron microscopic evaluation

For the scanning electron microscopic (SEM) evaluation, the remaining parts of the excised lingual tissues were fixed in 2% glutaraldehyde in cacodylate buffer for 2 h, followed by three washes in buffer for 10 min each. Samples were then dehydrated in a series of increasing ethanol concentrations (30, 50, 75, 80, 90, 95, and 100%) for 10 min each, and then stored in collecting solution of 0.2 M sodium cacodylate added to 4% sucrose and 4% glutaraldehyde [36]. Subsequently, samples were fixed on aluminium stubs using a double-sided adhesive carbon tape and were sputter-coated with gold in a vacuum machine (Quorum, Q150R S+, UK) and examined with a scanning electron microscope (ZEISS, EVO15, UK).

### Quantitative characterization of the phytochemical composition of *Annona muricata*

Phytochemical profiling was conducted using liquid chromatography-electrospray ionization-tandem mass spectrometry (LC-ESI-MS/MS) in accordance with previously reported approach [37]. The analysis was performed on an Exion LC™ AC system (SCIEX, USA) coupled to a SCIEX Triple Quad 5500 + MS/MS system (SCIEX, Singapore) equipped with an electrospray ionization (ESI) detector. Chromatographic separation was performed on a Poroshell 120 EC-C18 column (3.0 × 100 mm, 2.7 μm), maintained at 30 °C, using a mobile phase of (A) 0.1% formic acid in water and (B) acetonitrile (LC-MS grade), delivered at a flow rate of 0.4–0.5 mL/min and a 5 µL injection volume. A gradient elution was applied, initiated at 8% B, progressively increased to 45% B, and subsequently returned to the initial conditions.

Detection was performed in negative ionization mode under multiple reaction monitoring (MRM) with optimized source parameters (-4500 V ion spray voltage, 500 °C source temperature, 25 psi curtain gas, 9 psi collision gas, and 50 psi ion source gas 1 and 2). The analysis was performed at the National Research Center, Giza, Egypt.

### Statistical analysis

Quantitative analyses of tissue fungal burden and body weight were performed using *Jamovi* software (version 2.6; The Jamovi Project, 2024). Log-transformed CFU/g values were compared among the five groups (R, RC, RCN, RCA, and RCNA) and between the two time points (days 7 and 10) using two-way ANOVA. Data normality and variance homogeneity were evaluated with the Shapiro-Wilk and Levene’s tests, respectively. When significant effects were observed, pairwise comparisons were conducted using the Games-Howell post hoc test, and effect sizes were expressed as partial eta squared (η²). To ensure robustness, results were confirmed by a 20% trimmed means ANOVA. Statistical significance was defined as *p* < 0.05.

Body weight data were analyzed using a linear mixed-effects model (LMM) to assess the effects of treatment group, time (days 1–10), and their interaction. Group, time, and their interaction were modeled as fixed effects. Estimated marginal means with 95% confidence intervals were used for post hoc comparisons, and residual normality was verified using the Shapiro-Wilk and Kolmogorov-Smirnov tests.

## Results

The therapeutic potential of *Annona muricata* (*A. muricata*) in comparison with nystatin and their combination was evaluated in gamma-irradiated rats orally infected with *Candida tropicalis* (*C. tropicalis*). Five experimental groups were analyzed; irradiation-only group (R), infected irradiated (RC), rats treated with nystatin only (RCN), rats treated with *A. muricata* only (RCA), and rats treated with both *A. muricata* and nystatin (RCNA). The outcomes were assessed at the respective endpoints of each group (days 7 and 10).

### Morphological observations

**Fig. 2:**
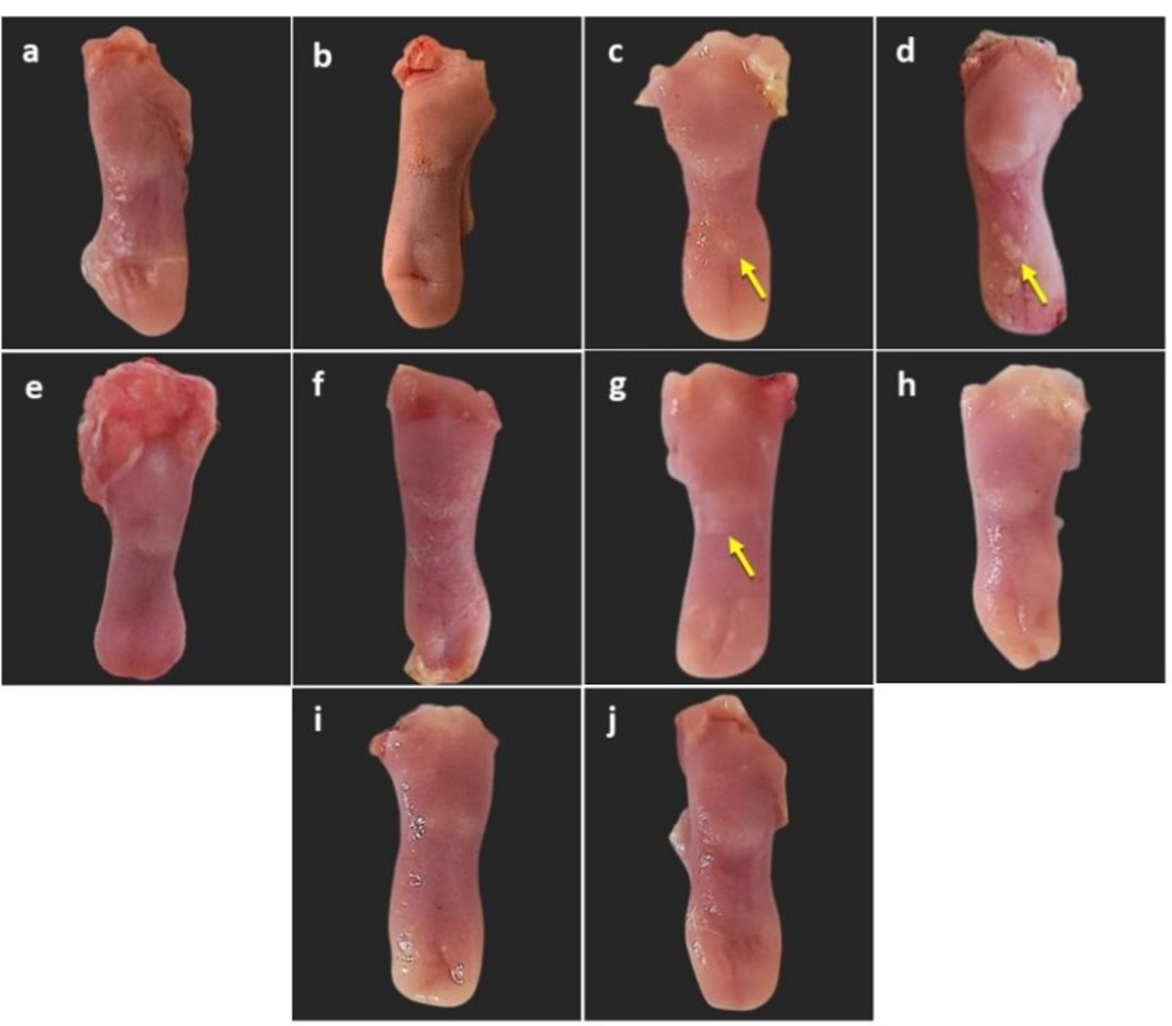
Gross morphology of gamma-irradiated rats’ tongues across experimental groups. **a**, **b** R group at days 7 and 10, respectively. **c**, **d** RC group at days 7 and 10, respectively, showing ulcerative lesions (yellow arrows) consistent with active candidiasis. **e**, **f** RCN group at days 7 and 10, respectively, with no ulcerative lesions, indicating preserved tissue integrity under therapeutic intervention. **g**, **h** RCA group at days 7 and 10, respectively, showing limited ulcer formation at day 7 (yellow arrow) with partial improvement and absence of ulcerative lesions by day 10. **i**, **j** RCNA group at days 7 and 10, respectively, with no ulcerative lesions observed, indicating maintenance of tissue integrity following therapeutic intervention

Gross morphological examination of the tongues revealed distinct differences among the experimental groups. In the irradiation-only group, the dorsal tongue surface appeared normal, with intact mucosa that was slightly dry and pale, with no evidence of ulceration or other tissue abnormalities. In the RC group, superficial ulcers were evident by day 7, which became more extensive by day 10. In contrast, the RCA group displayed only a few small ulcerative lesions at day 7, while by day 10 the tongues surfaces appeared intact with no detectable abnormalities. Notably, no visible pathological alterations were observed in the RCN or RCNA groups at either time points, indicating the effect of the respective treatments (Fig. 2).

### Body weight results

**Fig. 3:**
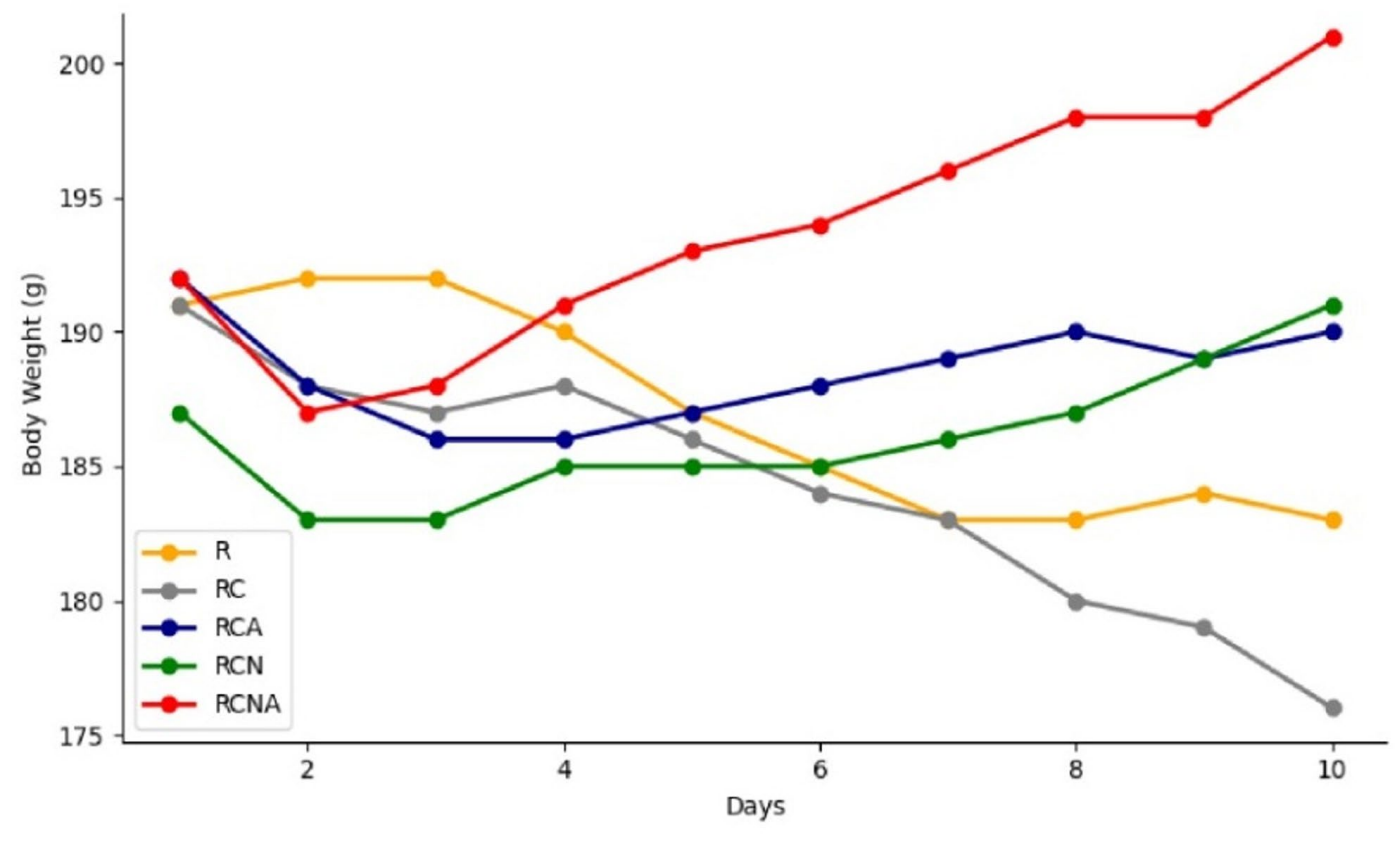
Mean body weight changes across experimental days in all groups

Body weight was daily monitored across experimental groups to evaluate the effect of *C. tropicalis* infection as well as different treatments on gamma-irradiated rats. A linear mixed effects model was applied. Groups (R, RC, RCN, RCA, and RCNA) and days (1–10) were considered as fixed effects to account for repeated measures within rats. An autoregressive residual structure was used for within-subject correlations over time (Fig. 3).

A significant main effect of group was detected (F = 3.66, *p* = 0.006), demonstrating overall differences in body weight between treatment groups. The main effect of day was highly significant (F = 10.87, *p* < 0.001), reflecting a progressive change in body weight over time. Importantly, there was a highly significant group × day interaction (F = 9.42, *p* < 0.001), indicating that the progressive pattern of weight change differed among groups.

Post-hoc pairwise comparisons of the estimated marginal means revealed that the RCNA group exhibited the highest mean body weight across the experimental period (194 ± 2.07 g), followed by RCA (188 ± 2.03 g), R (187 ± 2.04 g), RCN (186 ± 2.1 g), and the untreated infected RC group (184 ± 2.1 g). Starting from day 6, RCNA animals maintained significantly higher body weights than RC (*p* < 0.001), with differences exceeding 20 g by day 10. Both single-treatment groups (RCN and RCA) also showed partial improvement compared with RC, though less pronounced than the combination therapy. Rats in the irradiation-only group (R) maintained relatively stable body weight compared with the infected untreated RC group, suggesting that radiation alone did not account for the marked weight decline associated with candidiasis.

Overall, these findings suggest that *A. muricata* and nystatin each contributed to the restoration of normal weight in infected animals, and their combination produced a more pronounced improved effect against infection-associated weight loss compared to the infected untreated (RC) group and single treatment groups.

### Quantitative assessment of tissue burden

Fungal burden in tongue tissues was assessed to evaluate the extent of *C. tropicalis* colonization and the effect of different treatments across the experimental groups. The mean log_10_ CFU/g values showed a clear reduction from day 7 to day 10 across all treatment groups (Table 1). The irradiation-only group (R) showed no detectable fungal growth at either time points, confirming the absence of infection in non-inoculated animals.

**Table 1:** Quantitative assessment of fungal burden in tongue tissues of gamma-irradiated rats across all groups

Group	Log_10_ CFU	
	Day 7	Day 10
R	0.00 ± 0.00^a1^	0.00 ± 0.00^a1^
RC	6.45 ± 0.13^b1^	6.76 ± 0.06^b1^
RCN	5.86 ± 0.07^c1^	4.34 ± 0.13^c2^
RCA	6.10 ± 0.24^c1^	5.23 ± 0.20^d2^
RCNA	5.37 ± 0.24^d1^	2.47 ± 0.38^e2^

Data are expressed as mean ± standard deviation at days 7 and 10. Different superscript letters within the same column indicate significant differences between groups at the same time point (Tukey post hoc test, *p* < 0.001). Different superscript numbers within the same row indicate significant differences between the two time points in the same group. Two-way ANOVA demonstrated significant effects of treatment group (*p* < 0.001, η²p = 0.995), time (*p* < 0.001, η²p = 0.906), and their interaction (*p* < 0.001, η²p = 0.928)

Two-way ANOVA revealed a highly significant effect of treatment type (F = 1535.4, *p* < 0.001, η²*p* = 0.995), indicating that the fungal burden varied substantially among groups. There was also a strong effect of time (F = 288.6, *p* < 0.001, η²*p* = 0.906), confirming that CFU counts further declined after the end of the treatment.

A significant interaction between treatment and time (F = 96.0, *p* < 0.001, η²*p* = 0.928) showed that the pattern of reduction over time differed among treatments. Post hoc comparisons demonstrated that all groups differed significantly from each other (*p* < 0.001). The untreated infected group (RC) maintained the highest CFU counts, while the combination therapy (RCNA) retained the greatest reduction, followed by RCN and RCA. Due to the absence of fungal colonization in R group, it exhibited significantly lower CFU values compared to all infected groups.

**Fig. 4:**
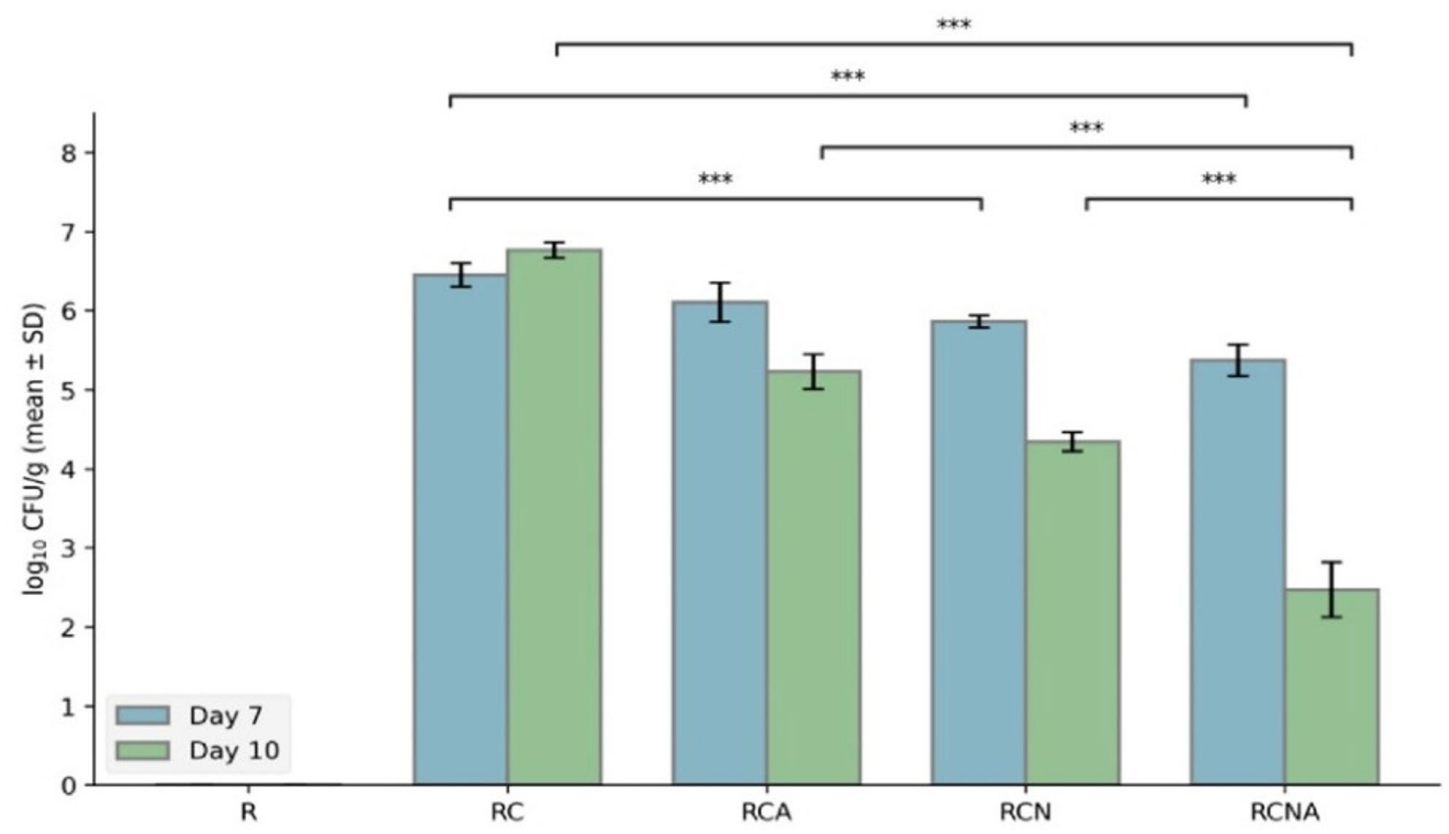
Mean ± SD of fungal tissue burden in each group at Days 7 and 10. Two-way ANOVA was used followed by Tukey post hoc comparisons. Significant effects were detected for treatment group (*p* < 0.001, η²p = 0.995), time (*p* < 0.001, η²p = 0.906), and the group × time interaction (*p* < 0.001, η²p = 0.928). *** means highly significant differences (*p* < 0.001)

Within-group comparisons showed that CFU levels significantly decreased between day 7 and day 10 in RCA, RCN, and RCNA (*p* < 0.001), whereas the RC group did not show a significant change over time (*p* = 0.377), suggesting that fungal clearance continued even after the end of treatment period (Fig. 4).

To ensure that these findings were not affected by unequal variances, a robust ANOVA using trimmed means was also performed. This analysis confirmed the same highly significant effects of treatment, time, as well as their interaction (*p* = 0.001). Together, these results indicate that all treatments were effective in lowering the fungal burden; with the combined treatment (RCNA) showing the most pronounced and sustained antifungal effect.

### Histopathological evaluation

**Fig. 5:**
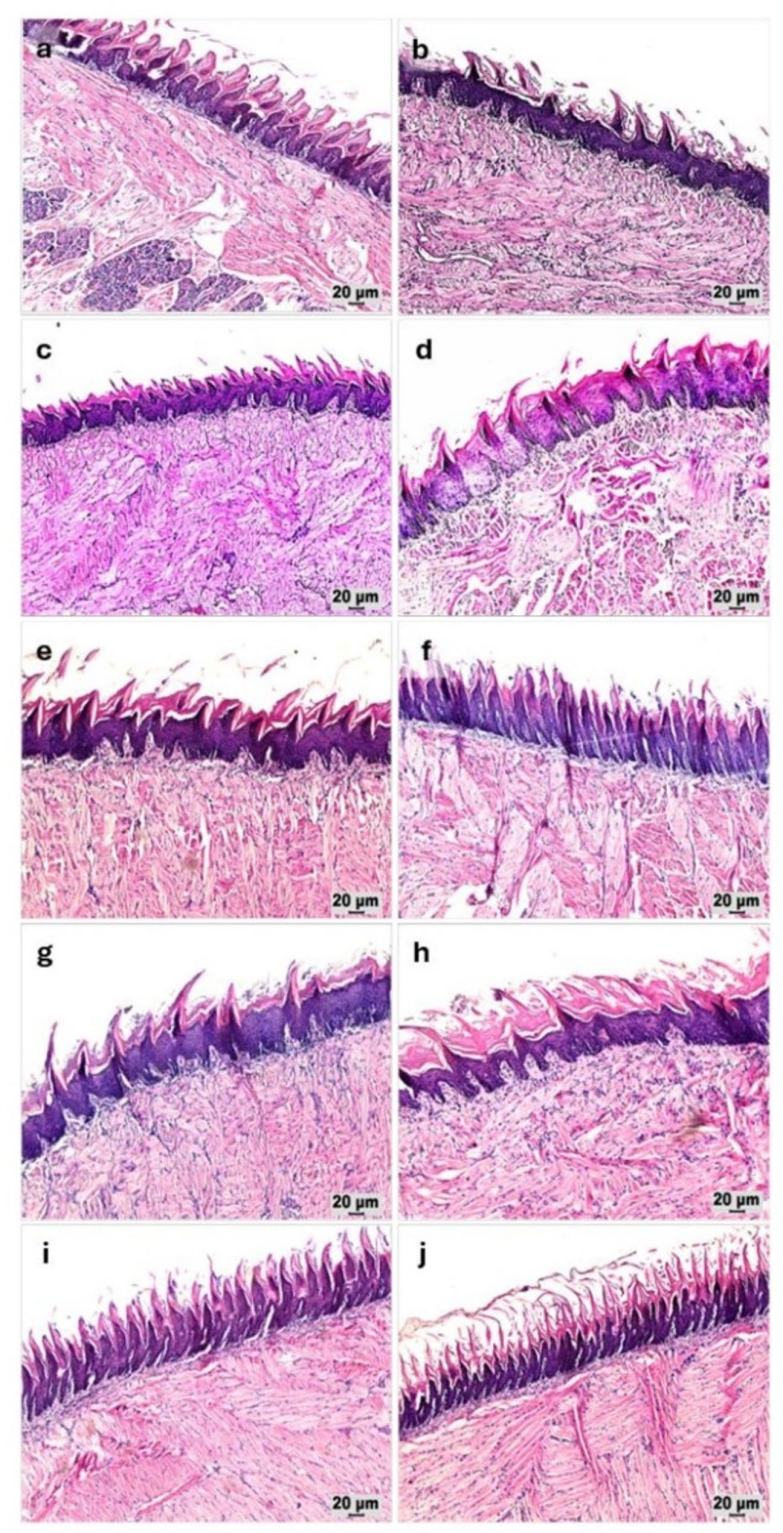
Photomicrographs of H&E-stained sections of gamma-irradiated rats’ tongues demonstrating overall epithelial and subepithelial architecture under different experimental conditions (100x). **a**, **b** R group at days 7 and 10, respectively. **c**, **d** RC group at days 7 and 10, respectively, demonstrating the most severe epithelial and connective tissue disruption. **e**, **f** RCN group at days 7 and 10, respectively, exhibiting marked improvement at day 7 and more evident restoration by day 10. **g**, **h** RCA group at days 7 and 10, respectively, showing mild improvement at day 7 with further structural recovery at day 10. **i**, **j** RCNA group at days 7 and 10, respectively, displaying the most preserved tissue integrity and architecture across both days 7 and 10

**Fig. 6:**
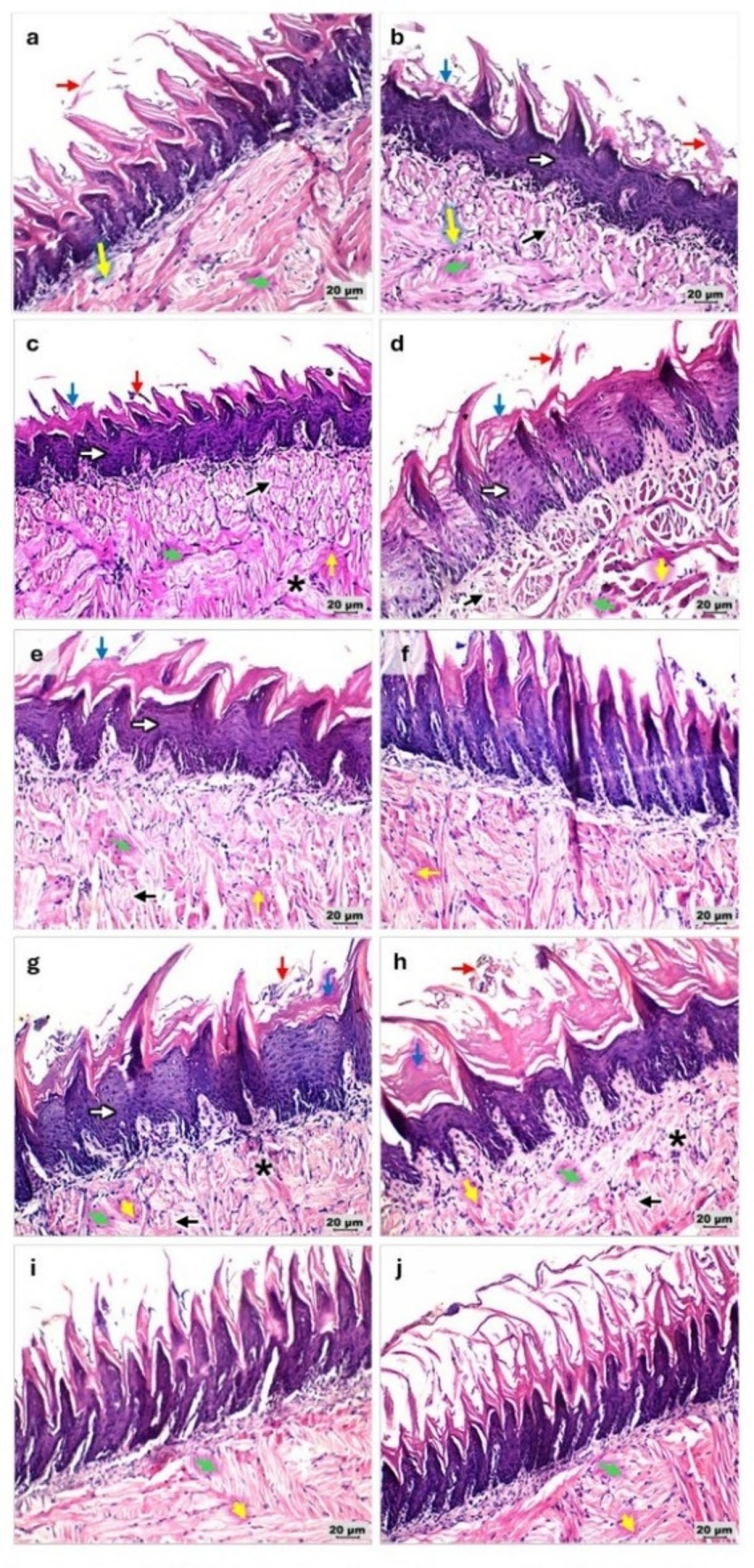
Photomicrographs of H&E-stained sections of gamma-irradiated rats’ tongues demonstrating histopathological alterations under different experimental conditions (200x). **a**, **b** R group at days 7 and 10, respectively. **c**, **d** RC group at days 7 and 10, respectively. **e**, **f **RCN group at days 7 and 10, respectively. **g**, **h** RCA group at days 7 and 10, respectively. **i**, **j** RCNA group at days 7 and 10, respectively. Green arrows; epithelial splitting, yellow arrows; pyknotic nuclei, white arrows; epithelial hyperplasia (acanthosis), black arrows; epithelial degeneration, red arrows; epithelial desquamation, blue arrows; hyperkeratosis, and asterisks; inflammatory cellular infiltration

Histopathological examination of tongue tissues using H&E and PAS staining was performed to assess epithelial integrity, inflammatory response, and the extent of fungal invasion. Sections of irradiation-only group (R) showed epithelial splitting, pyknotic nuclei, desquamation, and hyperkeratosis at day 7, with additional epithelial hyperplasia (acanthosis) and epithelial degeneration evident at day 10. Microscopic examination revealed progressive epithelial and subepithelial alterations in *Candida*-infected gamma-irradiated rats, with noticeable differences among treatment groups and time points (Figs. 5 and 6). In the RC group, epithelial splitting, degeneration, and marked desquamation were evident at day 7, accompanied by inflammatory cell infiltration. By day 10, these changes were more severe, with thinning of the epithelial layer, nuclear pyknosis, and connective tissue disruption.

Treatment with nystatin (RCN) led to marked improvement at day 7 characterized by partial epithelial restoration and reduced inflammatory infiltration. By day 10, tissue morphology showed near-normal architecture with diminished degenerative changes. The RCA group exhibited mild improvements at day 7, with persistence of hyperkeratosis and focal epithelial damage; however, by day 10, re-epithelialization and reduced inflammatory activity indicated progressive recovery. Notably, the RCNA group displayed the most preserved epithelial integrity across both time points, with minimal degeneration, limited hyperplasia, and markedly reduced inflammatory infiltration.

**Fig. 7:**
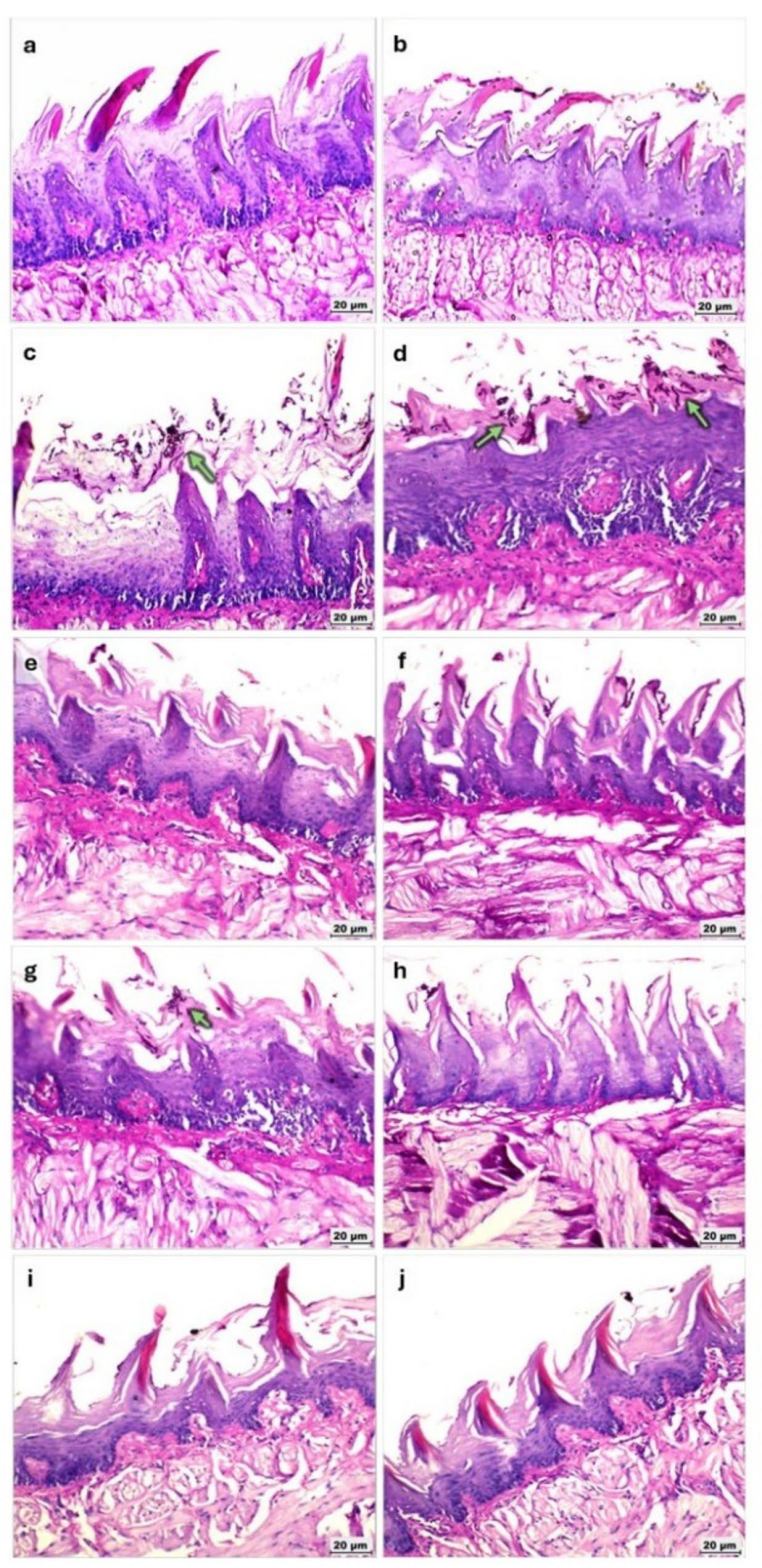
Photomicrographs of periodic acid–Schiff (PAS) stained sections of gamma-irradiated rats’ tongues demonstrating *Candida* infiltration under different experimental conditions (400x). **a**, **b** R group at days 7 and 10, respectively. **c**, **d** RC group at days 7 and 10, respectively. **e**, **f** RCN group at days 7 and 10, respectively. **g**, **h** RCA group at days 7 and 10, respectively. **i**, **j** RCNA group at days 7 and 10, respectively. Green arrows indicate Candida yeast and hyphal elements

PAS staining further confirmed the presence and distribution of fungal elements within the tongue tissues. In the RC group, abundant fungal elements were detected at both days 7 and 10, consistent with persistent infection and progressive tissue invasion. In the RCA group, fungal elements were observed at day 7 but were absent by day 10, indicating effective clearance over time. Importantly, no fungal structures were detected in the RCN or RCNA groups at either time points (Fig. 7).

### Scanning electron microscopic findings

**Fig. 8:**
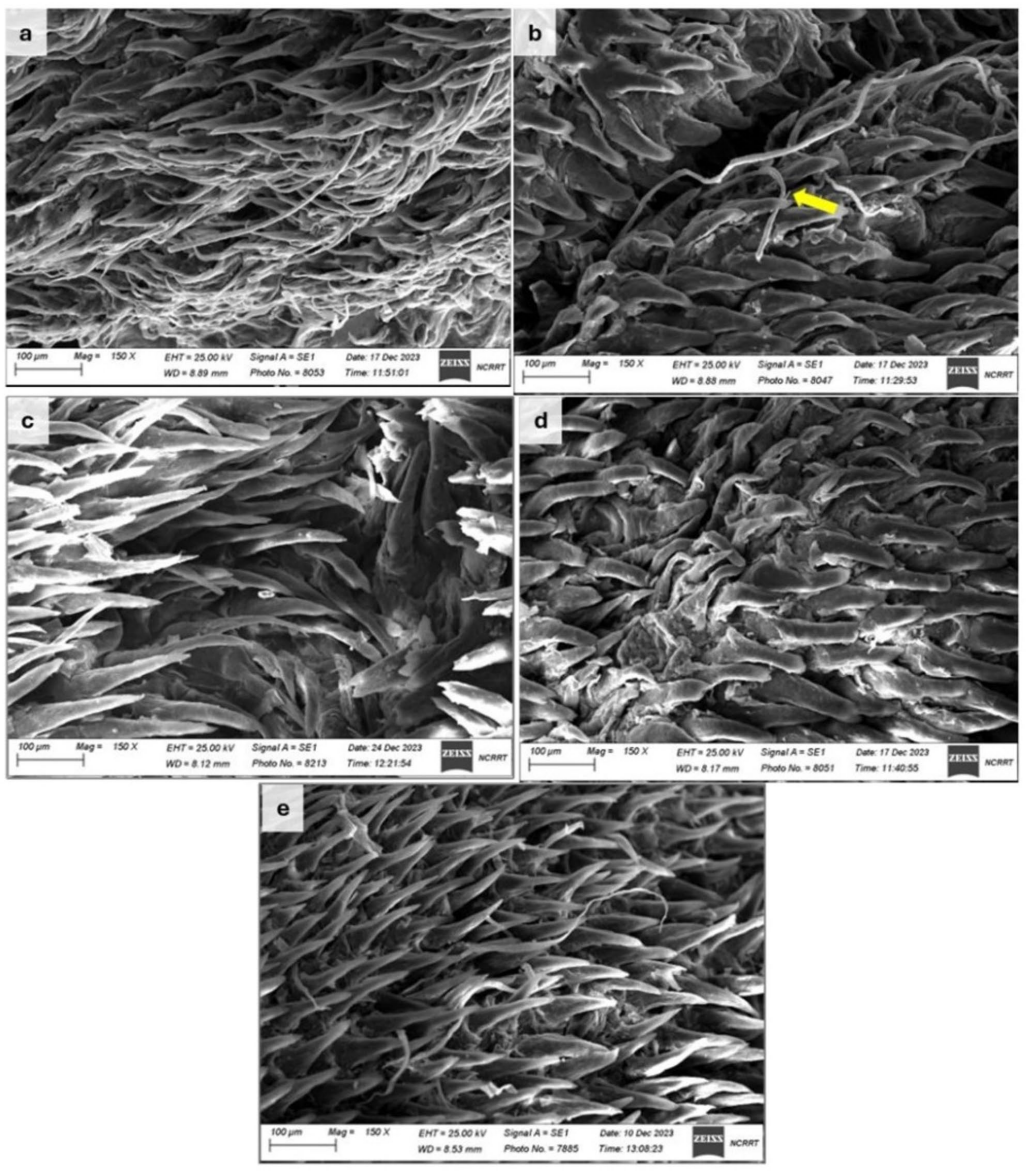
Scanning electron micrographs of the dorsal surface of gamma-irradiated rats’ tongues (150×). **a** R group reveals epithelial damage and papillary distortion. **b** RC group shows marked epithelial disruption, irregular compacted papillae, and prominent fungal hyphae (yellow arrow). **c** RCN group reveals substantial restoration of papillary alignment and surface architecture, with minimal disruption. **d** RCA group demonstrates partial improvement, with irregular papillae. **e** RCNA group displays the most preserved morphology, with elongated, well-organized papillae resembling near-normal epithelial structure

Scanning electron microscopy (SEM) was performed for tongue tissues at day 10. The earlier time point (day 7) was excluded from SEM analysis, as the histopathological and microbiological evaluations consistently indicated incomplete tissue repair and persistent fungal colonization at this point. Therefore, day 10 was selected as the representative endpoint for ultrastructural evaluation. In R group, epithelial damage and distortion of lingual papillae was observed. In the RC group, the epithelium appeared markedly disrupted, with disorganized papillae and visible hyphal projections, reflecting severe candidal invasion. SEM provided further confirmation of treatment-related differences in tongue surface morphology. Treatment with *A. muricata* (RCA) resulted in partial improvement, as papillae were more distinguishable but retained irregularities and roughness. In contrast, nystatin therapy (RCN) produced more pronounced recovery, with papillae exhibiting improved alignment and reduced surface damage. The greatest degree of preservation was observed in the combined treatment group (RCNA), where papillae appeared intact, elongated, and well organized, closely resembling the normal architecture of the tongue surface (Fig. 8).

### Quantitative phytochemical profiling of *Annona muricata*

**Fig. 9:**
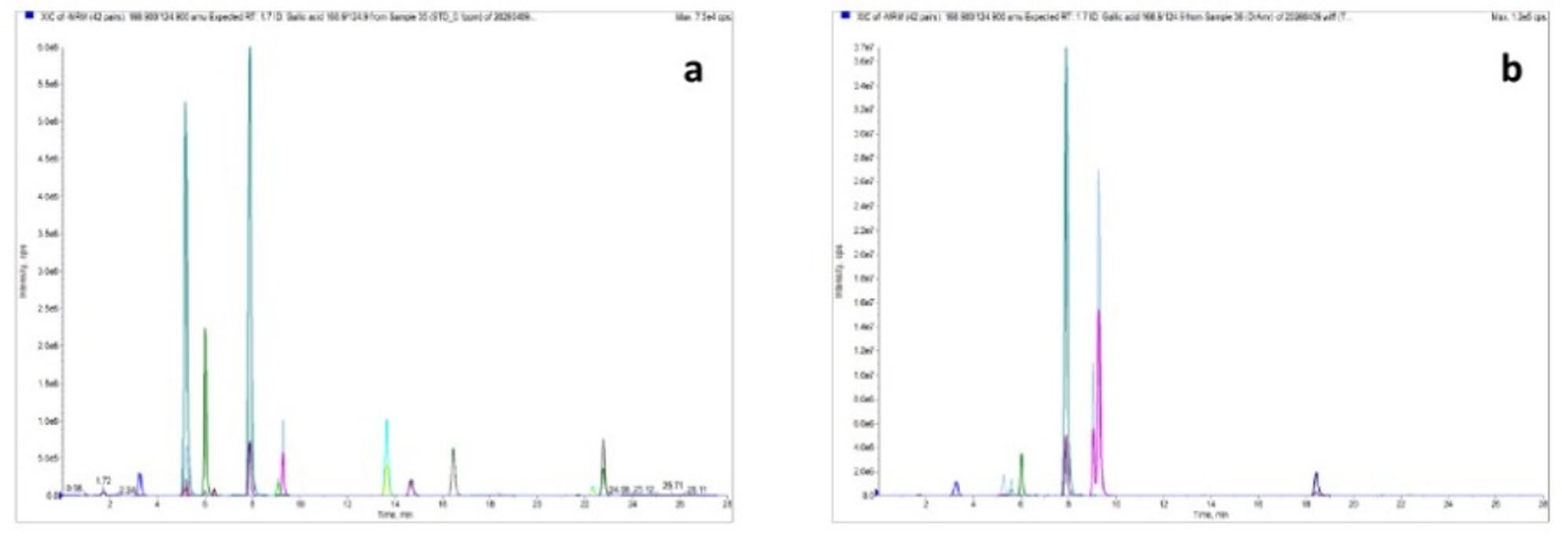
**a** Extracted ion chromatograms of mixture of phenolic and flavonoid standards (0.1 ppm). **b** Extracted ion chromatograms of *A. muricata* capsule (Graviola)

Quantitative LC-ESI-MS/MS analysis revealed the presence of 15 phenolic constituents, comprising five flavonoids and ten phenolic acids. The detected compounds showed considerable variation in their concentrations in the analyzed *A. muricata* capsule (Table 2, Fig. 9).

**Table 2:** Phytochemical constituents identified by LC-ESI-MS/MS and their concentrations in the analyzed A. muricata capsule

Compound	Concentration (ug/g)	Class
Major		
Rutin	294.07	Flavonoids
Naringenin	120.64	Flavonoids
Coumaric acid	75.09	Phenolic acids
3.4-Dihydroxybenzoic acid	44.86	Phenolic acids
Catechin	16.63	Flavonoids
Caffeic acid	14.71	Phenolic acids
Kaempferol	12.41	Flavonoids
Chlorogenic acid	11.10	Phenolic acids
Quercetin	10.06	Flavonoids
Minor		
Gallic acid	3.98	Phenolic acids
Ferulic acid	3.52	Phenolic acids
Syringic acid	3.12	Phenolic acids
Rosmarinic acid	0.29	Phenolic acids
Ellagic acid	0.23	Phenolic acids
Methyl gallate	0.01	Phenolic acids

Among the flavonoids, rutin was the predominant compound, exhibiting the highest concentration (294.07 µg/g), followed by naringenin (120.64 µg/g). The remaining flavonoids, including catechin (16.63 µg/g), kaempferol (12.41 µg/g), and quercetin (10.06 µg/g), were detected at comparatively lower levels.

Within the phenolic acids, p-coumaric acid represented the most abundant compound (75.09 µg/g), followed by 3,4-dihydroxybenzoic acid (44.86 µg/g). Moderate levels of caffeic acid (14.71 µg/g) and chlorogenic acid (11.10 µg/g) were also observed. Other phenolic acids, including gallic acid (3.98 µg/g), ferulic acid (3.52 µg/g), and syringic acid (3.12 µg/g), were detected in relatively smaller amounts, whereas rosmarinic acid (0.29 µg/g), ellagic acid (0.23 µg/g), and methyl gallate (0.01 µg/g) were present at trace levels.

**Fig. 10:**
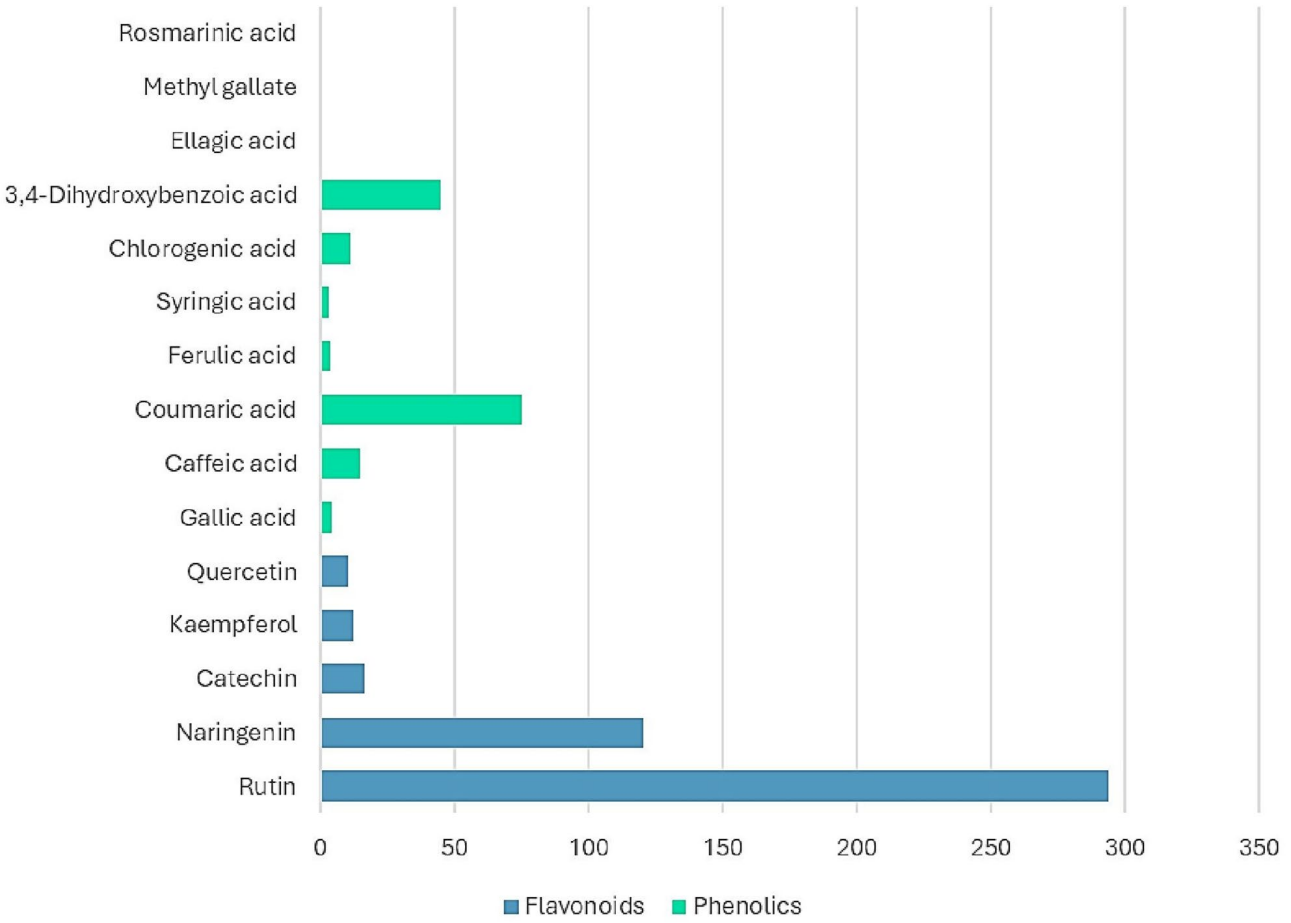
Quantitative LC-ESI-MS/MS analysis of the phytochemical profile of *A. muricata* showing the concentration (µg/g) of the identified compounds

Overall, flavonoids constituted the dominant phytochemical class, primarily due to the high abundance of rutin and naringenin, while the phenolic acids exhibited a broader but generally lower distribution of individual compounds (Fig. 10).

## Discussion

The present study aimed to evaluate the therapeutic efficacy of *Annona muricata* (*A. muricata*) compared with nystatin and their combination in managing oral *Candida tropicalis* (*C. tropicalis*) infection in gamma-irradiated rats, a model simulating the oral complications frequently encountered in head and neck cancer patients undergoing radiotherapy (RT).

Head and neck cancers represent a significant global health challenge, with over 890,000 new cases and about 450,000 deaths annually [38]. Their management typically includes surgery, chemotherapy, and RT, with RT remaining a cornerstone for achieving local tumor control [39].

Despite its therapeutic efficacy, RT causes oral complications by damaging epithelial integrity, reducing salivary flow, and impairing immune defenses, which facilitate microbial colonization and infection [11, 40]. Patients commonly develop oral mucositis and remain susceptible to secondary infections despite strict oral hygiene [41–43]. Consequently, effective management should not only target microbial suppression but also promote mucosal regeneration and epithelial repair.

The human oral cavity hosts a diverse microbiota, including bacteria, fungi, and viruses which establish colonization shortly after birth [44]. Among fungi, *Candida* species are the most common commensals, primarily found on the tongue’s dorsal surface, followed by the palate and buccal mucosa [41, 42, 45]. While typically part of the normal oral flora, *Candida* can become pathogenic when mucosal or immune barriers are compromised, such as during radiotherapy [38–40]. Evidences suggest that median rhomboid glossitis is predominantly initiated by *Candida* species [46].

Oral candidiasis is the most common opportunistic fungal infection in the mouth, often termed the “disease of the diseased” and occurs in individuals with underlying conditions such as diabetes, prolonged corticosteroid use, immunodeficiency, or cancer therapy [43, 47, 48]. Radiotherapy significantly increases *Candida* colonization; raising oral loads of *C. albicans* and other non-*albicans* species [41, 46, 47]. The rise in non-*albicans* species complicates diagnosis and management due to varied antifungal resistance [49]. Persistent infections may hinder treatment efficacy and, in severe cases, lead to systemic fungal spread [50, 51]. Furthermore, oral candidiasis may predispose patients to secondary bacterial infections, emphasizing the need for effective therapeutic approaches in immunocompromised individuals [43, 52].

Given its clinical relevance, oral candidiasis represents an important experimental model for investigating opportunistic fungal infections. Male rats are often selected for such studies owing to their ease of handling and the stable hormonal profiles that reduces variability associated with hormonal fluctuations [53]. The study focused on irradiated, infected rats to model clinical radiotherapy conditions. Non-irradiated controls were excluded due to spontaneous infection clearance and to follow ethical reduction principles (3Rs).

Noteworthy, *Candida* colonization patterns in rats differ from those in humans since it is not a natural component of the rat oral microbiota [54]. In the present study, a reproducible and uniform oral infection was successfully established in *Candida*-free rats, with clinical and histopathological evidence of lesion formation following oral inoculation of *C. tropicalis*. A single 20 Gy dose was used to simulate acute radiation exposure and assess early tissue and immune responses. Although clinical radiotherapy is fractionated, single-dose irradiation is widely used in preclinical studies [28] to reliably induce oral mucosal injury. Morphological assessment, body weight monitoring, quantitative tissue burden analysis, and scanning electron microscopy collectively confirmed the validity of the developed model. Daily body weight measurements were used only as a supportive parameter and not as a primary outcome measure or an endpoint. It acts as an indicator of oral pain and feeding behavior [6].

The irradiation-only group (R) maintained relatively stable body weight throughout the experiment and demonstrated no fungal colonization at either time points. Histopathological assessment revealed progressive epithelial damage characterized by splitting, nuclear pyknosis, desquamation, hyperkeratosis, and eventual epithelial hyperplasia (acanthosis) with evident degeneration. Collectively, these findings are indicative of radiation-induced mucosal injury occurring independently of superimposed infection. In the untreated infected irradiated group (RC), progressive oral lesions were evident throughout the experimental period. By day 7, the tongues exhibited superficial ulcerations that became extensive by day 10, accompanied by significant body weight loss. Histopathological examination revealed epithelial splitting, degeneration, and intense inflammatory infiltration, which worsened over time, leading to epithelial thinning and connective tissue disruption. Scanning electron microscopy (SEM) confirmed marked epithelial disorganization with visible hyphal elements, indicating severe and persistent *C. tropicalis* invasion without signs of recovery.

The obtained results confirm the ability of *C. tropicalis* to successfully establish oral infection in irradiated hosts, supporting its recognized pathogenic potential in radiotherapeutic patients. *C. tropicalis* has been identified as the second most virulent after *C. albicans* and the most frequently isolated species in patients receiving head and neck RT [42, 55]. It is also linked to a higher incidence of disseminated infection [51]. Its pathogenicity is mainly attributed to the production of lytic enzymes such as phospholipases and proteases [55–57], hyphal transformation, and strong biofilm formation, which together enhance adherence to mucosal surfaces and reduce antifungal susceptibility [55]. These virulence traits, when combined with radiation-induced mucosal injury and immunosuppression, can aggravate infection severity and hinder mucosal recovery [57].

In our study, histological examination using PAS stains demonstrates the presence of pseudohyphae penetrating the superficial epithelial layer of RC group. In a study conducted by Lauwers and co-authors [51], the degree and frequency of epithelial penetration differ among *Candida* species and even between strains. Notably, *C. tropicalis* was able to invade epithelial tissues in several experimental models. Furthermore, in the RC group, pronounced epithelial splitting, cellular degeneration, and extensive desquamation were observed, accompanied by a dense inflammatory cell infiltrate. Inflammatory reactions have been documented in both the epithelial and subepithelial tissues in experimental rat model of oral candidiasis, with occasional development of micro-abscesses [52]. In patients receiving head and neck RT, mucosal ulcerations resulting from oral mucositis may facilitate fungal invasion into underlying tissues, thereby intensifying interactions between *Candida* species and the connective tissue matrix [43].

The management of oral candidiasis remains a major clinical challenge [11]. Conventional antifungals, including polyenes, azoles, and echinocandins vary in their effectiveness. Nystatin, a broad-spectrum polyene macrolide active against yeast species including *C. tropicalis *[12], is mainly used for localized infections like oral candidiasis [58]. In this study, nystatin was used as the reference antifungal due to its widespread use, proven efficacy, and cost-effectiveness in treating oral candidiasis.

Our results demonstrated that treatment with nystatin markedly improved oral candidiasis-induced lesions in gamma-irradiated rats. Tongue gross morphology appeared normal, and fungal tissue burden was notably reduced compared to untreated infected rats. Histopathological examination revealed that at day 7, the epithelium showed partial restoration with reduced inflammatory infiltration, while by day 10, tissue architecture was nearly normal with minimal degenerative changes, indicating substantial healing and recovery.

The therapeutic effects of nystatin are consistent with its established mechanism of action, as it binds to ergosterol in fungal membranes, causing pore formation, ionic imbalance, and cell lysis [59]. This fungicidal property likely explains the absence of fungal elements and the nearly complete histological recovery observed in the treated RCN group. However, since nystatin lacks antioxidant and anti-inflammatory effects [60], its contribution to systemic recovery appears limited, reflected by weight stabilization rather than gain. Topical nystatin remains the recommended therapy for oral candidiasis by clinical practice guidelines, as it offers strong local action against *Candida* colonizing the oral mucosa [47].

Management of fungal infections in radiotherapeutic patients is challenging compared to other patients, as radiation profoundly influences both host tissues and fungal behavior. Gamma radiation can alter key virulence traits of *C. tropicalis *[61]. da Silva et al. [61] observed that irradiation increased extracellular matrix and biofilm production, features typically linked to increased antifungal resistance, highlighting the complex and unpredictable effects of radiation on fungal behavior and drug susceptibility. In addition to these radiation-induced changes, the extensive use of antifungals has further contributed to resistance among *Candida* species, reducing the effectiveness of conventional therapies. Moreover, RT-induced mucosal injury and xerostomia can diminish the efficacy of topical antifungals and delay mucosal healing [40, 62].

Despite these difficulties, some studies report preserved antifungal sensitivity under radiotherapy. Khozeimeh et al. [63], found that all *Candida* species isolated before and after radiotherapy were fully sensitive to nystatin. Similarly, Golestannejad et al. [64], demonstrated that *Candida* isolates from head and neck cancer patients, including *C. tropicalis*, remained fully sensitive to nystatin. Conversely, Kinkela Devčić et al. [65] observed no significant reduction in *Candida* colony counts in an in vivo study, suggesting variability across experimental conditions.

These challenges highlight the need for alternative or adjunctive therapeutic approaches that combine strong antifungal efficacy with the ability to support tissue repair and mitigate inflammation. Consequently, increasing attention has been directed toward natural, plant-derived compounds with promising antifungal properties [47, 65].

Medicinal plants have long been important to drug discovery, serving as sources of bioactive compounds with diverse therapeutic effects [66]. Despite their recognized antioxidant, antimicrobial, and anticancer properties, only a small fraction of plant species has been comprehensively studied, leaving their pharmacological potential largely unidentified [18]. Among these, *A. muricata* is traditionally used to treat parasitic, bacterial, and fungal infections, as well as hypertension, inflammation, diabetes, and cancer [18, 66].

In the present study, *A. muricata* treatment markedly reduced the fungal load on rats’ tongues compared to untreated infected group, supporting its potential antifungal efficacy. Consistent results were reported by Cesar et al. [67], who found that methanolic extracts of *A. muricata* exhibited significant antifungal activity against various *Candida* species, and by Pai et al. [18], who demonstrated strong fungicidal effects of the extract against oral *Candida* isolates at multiple concentrations. However, Abdel-Rahman et al. [68], observed that while *A. muricata* seed extract was active against both Gram-positive and Gram-negative bacteria, *C. albicans* showed resistance to its effects. This variation among studies may reflect differences in extract type, concentration, or tested strains. Furthermore, recent in vitro and in vivo investigations confirmed the antifungal efficacy of *A. muricata* extracts against *Candida* spp., showing reduced biofilm formation and attenuation of mucosal inflammation in animal models. Campos et al. [15, 19] also reported that *A. muricata* inhibited *Candida* proliferation and biofilm development while improving mucosal inflammatory responses in vulvovaginal candidiasis models. These findings may explain the antimicrobial and anti-inflammatory effects observed in the present study regarding the RCA group and the fungal clearance noted by day 10.

Evidence from in vitro studies has demonstrated that *A. muricata* possesses notable antimicrobial, anti-inflammatory, antineoplastic, and antioxidant properties [69]. Among its bioactive constituents, acetogenins (ACGs), which are unique to the Annonaceae family play a central role [70]. In addition, *A. muricata* is rich in alkaloids, flavonoids, and other phytochemicals with significant pharmacological potential [71]. ACGs inhibit mitochondrial NADH dehydrogenases in yeast cells, thereby disrupting cellular respiration and triggering apoptosis [70, 72]. Alkaloids and flavonoids further contribute to antifungal activity of *A. muricata *[15].

LC-ESI-MS/MS profiling of *A. muricata* revealed multiple phenolic acids and flavonoids, including major constituents as rutin, naringenin, coumaric acid, 3,4-dihydroxybenzoic acid, catechin, caffeic acid, kaempferol, chlorogenic acid and quercetin. Minor constituents as gallic acid, ferulic acid, syringic acid, ellagic acid, rosmarinic acid and methyl gallate were also detected. Phenolic acids and flavonoids have been reported to exert antifungal activity against *Candida* species through multiple mechanisms. These mechanisms include compromising fungal cell wall integrity and damaging the cell membrane, leading to increased permeability and leakage of intracellular contents. Moreover, they induce oxidative stress, interfere with biofilm formation, inhibit ergosterol biosynthesis, and suppress virulence factors [15, 73].

Phenolic acids such as gallic, caffeic, ferulic, syringic, coumaric, 3,4-dihydroxybenzoic, methyl gallate and rosmarinic acids primarily act via cell membrane disruption and induction of oxidative stress, leading to increased reactive oxygen species (ROS) generation and subsequent fungal cell damage. Several of these compounds also interfere with fungal metabolic processes and contribute to growth inhibition [73, 74]. Chlorogenic acid further enhances antifungal efficacy through anti-biofilm activity and metabolic interference, targeting structured microbial communities that are typically more resistant to treatment [75].

Flavonoids (polyphenols) including quercetin, rutin, kaempferol, naringenin, and catechin exhibit more targeted cellular effects, such as mitochondrial dysfunction, apoptosis induction, inhibition of hyphal formation, and suppression of virulence factors, in addition to biofilm inhibition and reduced adhesion [73, 76–78]. Quercetin, in particular, induces fungal apoptosis via mitochondrial damage, while naringenin and catechin impair adhesion and structural integrity of the fungal cell wall [77–79]. Ellagic acid contributes through antioxidant and supportive antifungal effects, enhancing overall activity [73, 77, 80].

The diversity of detected phytochemicals suggests a potential synergistic effect, where the combined action of these constituents may have enhanced the overall biological activity of *A. muricata*, explaining the observed improvement and the antifungal efficacy of *A. muricata* against *C. tropicalis*.

*Annona muricata* alone demonstrated moderate effects at day 7 but substantial epithelial and connective tissue recovery, nearly intact epithelium, and minimal inflammatory infiltration by day 10, indicating that *A. muricata* facilitated mucosal repair. This time-dependent improvement may reflect its role in enhancing tissue regeneration rather than immediate fungicidal activity. The antioxidant and anti-inflammatory properties of *A. muricata* are believed to play a crucial role in modifying mucosal injury and enhancing tissue regeneration. This therapeutic potential is particularly significant following irradiation, where excessive oxidative stress and inflammatory responses contribute to mucosal degeneration and delayed healing [17]. The polyphenolic compounds present in *A. muricata* possess potent free-radical-scavenging capacity, which may protect epithelial cells from radiation-mediated oxidative damage [81].

The present study demonstrates that both *A. muricata* and nystatin possess significant therapeutic efficacy against radiation-associated oral candidiasis induced by *C. tropicalis*, with their combination yielding the most pronounced therapeutic outcomes at both time points. Assessment of the tongue fungal burden revealed a significant reduction in fungal load in the group treated with both *A. muricata* and nystatin compared to all other groups. The enhanced effectiveness of the combined regimen was evident through superior epithelial healing and regeneration, greater fungal clearance, as well as better body weight recovery, suggesting a potential synergistic interaction.

Nystatin exerts a rapid fungicidal effect through direct disruption of the fungal cell membrane, effectively reducing the microbial burden during the early infection phase. In parallel, *A. muricata* enhances this antifungal action while providing complementary benefits through its potent antioxidant and anti-inflammatory properties. These properties mitigate oxidative stress, modulate host immune responses, promote epithelial repair and restore normal tissue architecture. This may act more slowly or indirectly than polyenes [72]. These integrated effects not only suppress fungal persistence but also accelerate mucosal healing and overall systemic recovery, as reflected by the notable improvement in body weight observed in the combination group. This combined therapeutic approach may be particularly beneficial in immunocompromised patients, where fungal eradication alone may not be sufficient for complete clinical recovery.

The RCNA group demonstrated the most favourable histopathological features, including preserved epithelial architecture, absence of ulceration, and minimal inflammatory infiltration. The RCNA group maintained the highest mean body weights, diverging from the RC group from day 6 onward. This indicates that the combined therapy most effectively preserved oral function and appetite while attenuating the systemic consequences of mucosal infection.

Collectively, in gamma-irradiated rats orally infected with *C. tropicalis*, treatment with either *A. muricata* or nystatin alleviated mucosal lesions and reduced fungal load, whereas their combined administration yielded the most pronounced improvements across all evaluated parameters, including gross morphology, body weight, CFU counts, histopathology, and SEM findings. The single-agent treatments produced moderate recovery, with nystatin showing slightly greater efficacy than *A. muricata* alone. In contrast, untreated infected group displayed persistent ulceration, elevated fungal counts, numerous PAS-positive yeast cells, extensive tissue damage, and severe epithelial disruption under SEM. Accordingly, these outcomes reject the null hypotheses, suggesting that *A. muricata* exerts significant antifungal activity and that its combined use with nystatin produces an enhanced effect against oral candidiasis in gamma-irradiated rats.

Nonetheless, several limitations of this study should be acknowledged. First, although the rat model provides valuable insights, it cannot fully mimic the complexity of the human oral cavity and systemic immune responses. Second, the experimental design was limited to short-term endpoints (day 7 and day 10); longer-term investigations are required to evaluate recurrence and the maintenance of mucosal integrity. Lastly, the comparison between local and systemic antifungal treatments is limited by inherent differences in pharmacokinetics, bioavailability, and mechanisms of action, which may influence outcomes. Despite these limitations, this model serves as a practical and well controlled platform for evaluating different therapeutic approaches for managing oral candidiasis in radiotherapeutic head and neck cancer patients.

Future investigations should aim to clarify the molecular mechanisms responsible for the antifungal and host-protective effects of *A. muricata*. Moreover, studies should assess oxidative stress biomarkers, pro-inflammatory cytokine profiles, and epithelial regeneration pathways. Furthermore, alternative delivery systems, such as mouth rinses, gels, or nanoparticle-based formulations should be explored to improve bioavailability and patient compliance. Additionally, clinical trials in head and neck cancer patients undergoing radiotherapy are essential to confirm efficacy and safety in humans. The absence of molecular data highlights the need for further mechanistic studies.

## Conclusions

In our experiment, nystatin demonstrated effective antifungal activity, suggesting that it remains a reliable therapeutic option even in patients undergoing radiotherapy. However, incorporating *Annona muricata* could offer additional advantages, as its antioxidant and anti-inflammatory properties may support mucosal healing and provide systemic benefits, particularly in radiotherapeutic patients whose bodies experience considerable physiological stress from the disease or its treatment.

## Data Availability

All data generated or analyzed during this study are included in this published article.
